# LC–MS/MS-based in vitro and in vivo investigation of blood–brain barrier integrity by simultaneous quantitation of mannitol and sucrose

**DOI:** 10.1186/s12987-020-00224-1

**Published:** 2020-10-14

**Authors:** Behnam Noorani, Ekram Ahmed Chowdhury, Faleh Alqahtani, Yeseul Ahn, Dhavalkumar Patel, Abraham Al-Ahmad, Reza Mehvar, Ulrich Bickel

**Affiliations:** 1 Department of Pharmaceutical Sciences, Jerry H. Hodge School of Pharmacy, Texas Tech University Health Sciences Center, Amarillo, TX 79106 USA; 2 Department of Pharmacology and Toxicology, College of Pharmacy, King Saud University, Riyadh, 11451 Saudi Arabia; 3 Department of Biomedical and Pharmaceutical Sciences, Chapman University, School of Pharmacy, Irvine, CA USA; 4 Center for Blood–Brain Barrier Research, School of Pharmacy, Texas Tech University Health Sciences Center, Amarillo, TX 79106 USA

**Keywords:** Blood–brain barrier, Mannitol, Sucrose, Vascular space correction, Permeability coefficient, Brain uptake clearance, In vitro and in vivo correlation

## Abstract

**Background:**

Understanding the pathophysiology of the blood brain–barrier (BBB) plays a critical role in diagnosis and treatment of disease conditions. Applying a sensitive and specific LC–MS/MS technique for the measurement of BBB integrity with high precision, we have recently introduced non-radioactive [^13^C_12_]sucrose as a superior marker substance. Comparison of permeability markers with different molecular weight, but otherwise similar physicochemical properties, can provide insights into the uptake mechanism at the BBB. Mannitol is a small hydrophilic, uncharged molecule that is half the size of sucrose. Previously only radioactive [^3^H]mannitol or [^14^C]mannitol has been used to measure BBB integrity.

**Methods:**

We developed a UPLC–MS/MS method for simultaneous analysis of stable isotope-labeled sucrose and mannitol. The in vivo BBB permeability of [^13^C_6_]mannitol and [^13^C_12_]sucrose was measured in mice, using [^13^C_6_]sucrose as a vascular marker to correct for brain intravascular content. Moreover, a Transwell model with induced pluripotent stem cell-derived brain endothelial cells was used to measure the permeability coefficient of sucrose and mannitol in vitro both under control and compromised (in the presence of IL-1β) conditions.

**Results:**

We found low permeability values for both mannitol and sucrose in vitro (permeability coefficients of 4.99 ± 0.152 × 10^−7^ and 3.12 ± 0.176 × 10^−7^ cm/s, respectively) and in vivo (PS products of 0.267 ± 0.021 and 0.126 ± 0.025 µl g^−1^ min^−1^, respectively). Further, the in vitro permeability of both markers substantially increased in the presence of IL-1β. Corrected brain concentrations (C_br_), obtained by washout vs. vascular marker correction, were not significantly different for either mannitol (0.071 ± 0.007 and 0.065 ± 0.009 percent injected dose per g) or sucrose (0.035 ± 0.003 and 0.037 ± 0.005 percent injected dose per g). These data also indicate that C_br_ and PS product values of mannitol were about twice the corresponding values of sucrose.

**Conclusions:**

We established a highly sensitive, specific and reproducible approach to simultaneously measure the BBB permeability of two classical low molecular weight, hydrophilic markers in a stable isotope labeled format. This method is now available as a tool to quantify BBB permeability in vitro and in vivo in different disease models, as well as for monitoring treatment outcomes.

## Introduction

The blood–brain barrier (BBB) maintains the homeostatic environment of the CNS by separating circulating blood from the central nervous system [1]. It encompasses specialized endothelial cells with a basal lamina that supports the abluminal surface of the endothelium along with other supporting cells, such as pericytes, astrocytes, and neurons [1]. The brain microvascular endothelial cells with tight junctions and transporter proteins are the primary and main gatekeepers for the transportation of nutrients and metabolites, and for the efflux of neurotoxins [2, 3]. The BBB dysfunction and breakdown contribute to neurological disorders due to the transfer of harmful blood components into the brain, irregular transport, and dysregulated clearance of metabolites associated with reduced cerebral blood flow [4]. Therefore, measuring the functional integrity of BBB by various methods such as paracellular markers is frequently performed in in vitro and in vivo studies.

There are many technical and conceptual pitfalls associated with the experimental application of supposedly paracellular markers and the subsequent interpretation of data. One important aspect, which deserves mentioning, is the fact that these markers can serve two distinct purposes. The first purpose is that, due to their characteristically low BBB permeability, these substances are often used as so-called vascular markers. This is commonly the case when other, more permeable agents are measured in the same study. When used as a vascular marker, it is assumed that neglecting the extent of brain uptake of the substance during a short experimental time period (1 minute or less) does not significantly compromise the study. Therefore, any concentration measured in whole brain tissue presumably represents brain intravascular space (with reference to concentration in whole blood), or brain plasma volume (with reference to plasma concentration). Such intravascular space values can then be used to correct brain concentrations of other substances, before calculating their BBB permeability. The second purpose is to determine the genuine permeability values of the BBB markers themselves, which is not zero. The latter measurement, of course, also requires proper correction for intravascular volume. Major damage to the BBB, caused by severe disease processes, such as stroke or a relapse phase of multiple sclerosis, may be readily detected using various imaging techniques and a range of markers. However, for the quantification of subtle BBB impairment, markers with naturally low permeability, such as sucrose or mannitol, are used, because even a minor degree of barrier damage is expected to have a noticeable effect on their permeability. Such damage has been observed in acute situations, for instance caused by peripheral inflammatory pain [5, 6], and after major surgery, where it has been connected to the occurrence of postoperative cognitive dysfunction in animal studies [7] and in patients [8]. Subtle BBB damage has also been postulated to play a role in the pathophysiology of chronic diseases like Alzheimer’s dementia [9] or small vessel disease [10]. However, there is still uncertainty, as functional BBB changes related to drug transport could not be confirmed in animal models of Alzheimer’s disease [11, 12].

Radiolabeled versions of sucrose, in particular [^14^C]sucrose, have long been used as low molecular weight, hydrophilic markers. We have recently introduced [^13^C_12_]sucrose as a superior marker substance, which is non-radioactive and can be quantified by a sensitive and highly specific LC–MS/MS technique [13, 14]. The disaccharide sucrose may be considered as the most widely accepted standard for the precise measurement of paracellular BBB permeability due to its properties, such as being uncharged, absence of protein binding, and metabolic stability in the circulation [15]. Our lab has focused on understanding the uptake mechanism at the BBB of different molecular weight markers, which have similar physicochemical properties. Mannitol is a small molecule that is about half the size of sucrose and has otherwise similar characteristics as sucrose as a marker for the BBB. It also has a molecular weight (182 Da) in the range of many small-molecule drugs.

Furthermore, mannitol has been widely used over the last 30 years in the lactulose/mannitol (L/M) test as a common dual-sugar test to assess the intestinal barrier function [16]. The radiotracer version of mannitol has been used for measurement of BBB integrity, but it requires a radioactive license and special handling skills [17–22]. We have also shown that using the radiolabeled versions of a marker, in particular [^14^C]sucrose, might result in a substantial overestimation of the true BBB permeability due to the presence of low level lipid-soluble impurities in the radiolabeled versions of the marker.

The first objective of the present study was to develop a UPLC–MS/MS method, which allows simultaneous analysis of stable isotope-labeled sucrose and mannitol. The second objective was to show the application of these markers in BBB in an vitro and in vivo model. The application of a stable isotope-labeled version of mannitol as a marker for BBB has not been reported yet. Different stable isotope-labeled versions of mannitol and sucrose, respectively, are commercially available. The variants of each marker coelute from a BEH-amide UPLC column, but are separate from each other. This allowed the simultaneous use of both markers for BBB permeability analysis. Furthermore, for the first time, one variant of [^13^C]sucrose was used to correct vascular space for mannitol and sucrose simultaneously. Thus, we selected a suitable combination of mass transitions and settings of the mass detector to detect and quantify [^13^C_6_]mannitol and [^13^C_12_]sucrose as permeability markers, [^2^H_8_]mannitol and [^2^H_2_]sucrose as internal standards, and [^13^C_6_]sucrose as a vascular marker. Our method offers novel accurate biomarkers of different sizes for permeability measurements of the BBB in the preclinical phase.

## Methods

### Chemicals and reagents

[^13^C_6_]mannitol, [^2^H_8_]mannitol, [^13^C_12_]sucrose, [^13^C_6_]sucrose, and [^2^H_2_]sucrose were obtained from Omicron Biochemicals (South Hill Street, South Bend, IN, USA). LC–MS grade water was purchased under the brand name J.T. Baker from Avantor Performance Materials, Inc. (Center Valley, PA). LC–MS/MS grade acetonitrile, water, and analytical grade ammonium hydroxide were purchased from Fisher Scientific (Fair Lawn, NJ, USA). For anesthesia, isoflurane was purchased from Lloyd Laboratories (Shenandoah, IA, USA). Heparin solution was purchased from APP Pharmaceuticals (Schaumburg, IL, USA). All other chemicals were analytical grade and obtained from commercial sources.

### Mass spectrometric and chromatographic conditions

Analytes were detected using an AB SCIEX QTRAP^®^ 5500 triple quadrupole mass spectrometer (MS) attached to a Nexera UPLC system (Shimadzu Corporation). The UPLC system contained an autosampler (Sil-30AC), pumps (LC-30AD), a controller (CBM-20A), a degasser (DGA-20A5), and a column oven (CTO-30A). Analyst software was used for data acquisition and quantification. Chromatographic separation was performed using an Acquity B.E.H. amide (2.1 mm × 50 mm, 1.7 μm; Waters, Milford, MA, USA), attached to an inline filter with a pore size of 0.2 μm as a pre-column. The isocratic elution was acetonitrile: water: ammonium hydroxide (73:27:0.1, v/v), at a flow rate of 0.2 mL/min. The column temperature was maintained at 45 °C, and the autosampler was at 4 °C. The total run time was 6 min. However, MS data were collected from 1 to 4.5 min only, and the valve was diverted to waste before and after that time. Electrospray ionization with multiple reactions monitoring system in negative mode was used for the ionization source. The mass spectrometer parameters for [^13^C_12_]sucrose, [^13^C_6_]sucrose and [^2^H_2_]sucrose were optimized in our previous study [13], however, [^13^C_6_] and [^2^H_2_]sucrose mass spectrometer parameters were changed due to presence of an interfering peak in the blank plasma and brain samples at the same retention time when combined with mannitol transitions. The mass spectrometer conditions for [^13^C_6_] and [^2^H_8_]mannitol were optimized to get optimum M−H^−1^ signal by continuous infusion of 100 ng/ml mannitol solution with an infusion pump. The optimized mass spectrometer parameters were as follows: ion spray voltage, − 4500 V; collision gas, high; curtain gas, 30 psi; temperature, 600 °C; ion source gas 1 (nebulizer gas), 55 psi; and ion source gas 2 (turbo gas), 55 psi.

For [^13^C6] and [^2^H_8_]mannitol, the m/z transitions 187 → 92 and 189 → 73 were selected, respectively. Also, the transitions 353 → 92, 347 → 179 and 343 → 71 were used for [^13^C_12_], [^13^C_6_] and [^2^H_2_]sucrose.

### Standard curve preparation

Stock solutions of triple analytes [^13^C_6_] mannitol, [^13^C_12_], and [^13^C_6_]sucrose were prepared in water at a concentration of 10 mg/mL. Plasma standard curves were made by adding blank mouse plasma to stock solutions to get plasma concentrations of 1–100 μg/mL. Then, each concentration was diluted 100-fold in water to obtain specific plasma calibration standards of 10–1000 ng/mL. For brain standard curve, blank brain tissue was homogenized in water (1:19), and triple analytes were spiked into the homogenized brain. Homogenate concentrations ranging from 5 to 400 ng/mL were prepared by serial dilution.

### Sample preparation

For the deproteination process, all samples were diluted tenfold in acetonitrile: water (80:20) containing 20 ng/mL of [^2^H_2_]sucrose and [^2^H_8_]mannitol. Then, precipitated samples were vortexed and centrifuged at 20,000*g* for 10 min. The supernatant was transferred into autosampler inserts, and a sample volume of 5 μL was injected into the UPLC column.

### Method validation

#### Selectivity

Blank matrix samples from mice containing no analyte were run to obtain the selectivity of the method. Also, to ensure that there is no interference between analyte transitions, neat samples of single analytes without matrix were run.

#### Accuracy and precision

Inter and intra-day runs were performed to determine the accuracy and precision of the method. The quality control samples (low, medium, and high concentrations) were evaluated against calibration curves. The accuracy was calculated as a percentage of measured concentration over nominal concentration. Precision was calculated as a percentage of relative standard deviations (RSD). The acceptable inter and intra-run limits for the accuracy were set at 85–115% for the middle and high concentrations and 80–120% for the low concentration. The standard precision values were 15% (medium and high concentrations) or 20% (low concentration).

#### Linearity

The linearity of calibration curves was evaluated by the coefficient of determination (r^2^) of the linear regression analysis of the concentration–response data using a weight of 1/x, where x is the concentration. Weighting by 1/x is superior to analysis with equal weights, on the strength of higher accuracy and less variability at low concentrations.

#### Recovery

The recovery of triple analytes was calculated in diluted plasma and homogenized brain. We expected similar recovery of the sucrose analytes as mentioned in the results described previously [13, 14] since all of the analytes are stable labeled isotopes of the same chemical entity. Three concentrations representing low, medium, and high were selected from the calibration curve. In case of plasma matrix, 10, 100, and 1000 ng/mL were used, and 5, 50, and 400 ng/mL were selected for the brain homogenate. Five replicate samples were prepared in each of the respective matrices, as well as samples with equivalent concentrations in water as reference. The samples and references were subjected to the sample preparation method described above, and the peak areas of analytes were determined. Recovery was calculated as the percent of the ratio of peak areas Sample/Reference, where sample refers to the matrix and reference to water (neat sample), respectively.

#### Freeze–thaw stability

The freeze–thaw stability was performed by subjecting two neat concentrations of analytes (50 and 500 ng/mL) to three freeze/thaw cycles (n = 3). Prepared samples were stored at − 80 °C and thawed at room temperature for 1 h, in order to replicate the experimental conditions. The concentration of the analytes in the neat samples was compared to the standard curve.

#### Long-term stability

Long term storage stability of different isotope of sucrose was checked in our previous study [13]. In this section, long term stability of [^13^C_6_]mannitol was evaluated for the diluted plasma samples and brain homogenate at − 80 °C. Quality control samples at low, medium and high concentrations of analyte in the brain and plasma (n = 3) were stored at − 80 °C for 2 months. The stored samples were then compared against standards in order to assess stability.

### In vivo application of the method

Two groups of anesthetized C57BL/6J mice were used to perform the pharmacokinetic study. 8–10 weeks old female C57BL/6J mice with 23–27 g bodyweight were purchased from Jackson Laboratories (Bar Harbor, ME, USA). The experimental protocols were approved by the Institutional Animal Care and Use Committee at Texas Tech University Health Sciences Center and followed current NIH guidelines. A silicone face mask was used to apply isoflurane (4% for induction, 1.5–2% v/v for maintenance) in 70% nitrous oxide/30% oxygen at a flow rate of 1 L/min. By skin incisions, the jugular veins were exposed bilaterally at the neck for IV injections and blood sampling, respectively. [^13^C_6_]mannitol and [^13^C_12_]sucrose (10 mg/kg) were co-injected as an IV bolus dose into the jugular vein. Then, at 1, 5, 10, 20, and 30 min after injection, blood samples (40 µL) were collected from the contralateral jugular vein. The samples were used to generate plasma concentration–time curves in each individual animal. Two groups of animals were used to investigate the effect of [^13^C_6_]sucrose application as vascular marker compared to transcardiac perfusion for vascular space correction (washout group). In the washout group (n = 6), the thorax was opened immediately after the last time point of sampling (30 min), and 20 mL phosphate buffered saline (pH 7.4) at room temperature, was used to perform the vascular perfusion via the left ventricle of the heart (flow rate of 2 mL/min) using a Harvard syringe pump. In order to facilitate the outflow of blood from the brain and to visually confirm the complete blood removal from the brain following perfusion, both jugular veins were cut open at the start of perfusion. In the second group of the animals (n = 6), a bolus dose of the vascular marker [^13^C_6_]sucrose (10 mg/kg in saline) was injected intravenously 30 s before the last sampling time point. Afterwards, the animals were euthanized by decapitation. Collected blood samples were centrifuged, and supernatant plasma was separated for further analysis. Meninges were removed from the collected brains, and the forebrains were weighted without olfactory bulbs, cerebellum, or brain stem. Then, the brain and plasma samples homogenized and diluted respectively according to the sample preparation steps described in UPLC-MS/MS section and the homogenized brain and diluted plasma were stored at − 80 °C until measurement by the UPLC-MS/MS system.

The value of corrected brain concentration ($C_{br - corr}^{Analyte}$) in the vascular marker group, which received [^13^C_6_]sucrose, was determined as follows:

1 $$ C_{br - corr}^{Analyte} = \frac{{(V_{d} - V_{0} ) \times C_{pl}^{Analyte} }}{{1 - V_{0} }} $$

Here, *V*_*d*_ is the apparent volume of distribution of the BBB permeability marker, [^13^C_6_]mannitol and [^13^C_12_] sucrose, *V*_*0*_ is the apparent volume of distribution of the vascular marker, [^13^C_6_] sucrose, and $C_{pl}^{analyte}$ is the terminal (30 min) plasma concentration of [^13^C_6_]mannitol or [^13^C_12_]sucrose. *V*_*d*_ and *V*_*0*_ values were obtained using the following two equations.

2 $$ V_{d} = C_{br}^{analyte} /C_{pl}^{analyte} $$

3 $$ V_{0} = C_{br}^{vascular\,marker} /C_{pl}^{vascular\,marker} $$

where $C_{br}^{analyte}$ is the total uncorrected brain concentration of [^13^C_6_] mannitol or [^13^C_12_]sucrose and $C_{br}^{vascular\,marker}$ is the total (uncorrected) brain concentrations of [^13^C_6_]sucrose, at the terminal sampling time (30 min), and $C_{pl}^{vascular\,marker}$ is the terminal plasma concentration of the vascular marker at 30 min.

Brain tissue concentration values in the washout group, which had undergone buffer washout, were considered as corrected for intravascular content. Values for brain uptake clearance, K_in_, also known as the permeability-surface area product, were calculated using the following equations based on either uncorrected ($C_{br}^{analyte}$) or corrected ($C_{br - corr}^{analyte}$) brain concentrations of mannitol and sucrose:

4 $$ K_{in} = C_{br}^{analyte} /AUC_{0}^{T} $$

5 $$ K_{in - corr} = C_{br - corr}^{analyte} /AUC_{0}^{T} $$

where *AUC*_*0*_^*T*^ denotes the area under the plasma concentration–time curve from time point 0 to the terminal sampling time (30 min) for [^13^C_6_]mannitol and [^13^C_12_]sucrose. *AUC*_*0*_^*T*^ was estimated via the linear-logarithmic trapezoidal method.

For a comparison between in vitro and in vivo models, the K_in_ values or permeability surface area products (PS) were converted to permeability coefficients, taking 120 cm^2^/g of brain as the surface area of the BBB in vivo [23].

### In vitro application of the method

#### iPSCs differentiation to BMECs

IMR90-c4 induced pluripotent stem cell line was used from the WiCell cell repository (WiCell, Madison, WI, USA). iPSCs were differentiated into brain microvascular endothelial cells (BMECs) following the established protocol [24, 25]. Undifferentiated stem cells were seeded on six well tissue culture treated plates coated with matrigel (C-Matrigel; Corning, Corning, MA, USA) in Essential 8 medium (E8 Thermo Fisher, Waltham, MA, USA) containing 10 μM Y-27632 (Tocris, Minneapolis, MN, USA) at a density of 100,000 cells/mL. Cells were maintained in E8 for 3 days prior to differentiation. Then, differentiation was initiated using unconditioned medium [UM: Dulbecco’s modified Eagle’s medium/F12 with 15 mM HEPES (Thermo Fisher, Waltham, MA, USA), 20% knockout serum replacement (Thermo Fisher, Waltham, MA, USA), 1% non-essential amino acids (Thermo Fisher, Waltham, MA, USA), 0.5% Glutamax (Thermo Fisher, Waltham, MA, USA) and 0.1 mM β-mercaptoethanol (Sigma-Aldrich, St. Louis, MO, USA)] and maintained for 6 days. After 6 days, cells were incubated for two days with EC++ media [human serum-free endothelial medium (hESFM, Thermo Fisher, Waltham, MA, USA) supplemented with 1% bovine platelet-poor plasma-derived serum (PDS, Alfa Aesar, Ward Mill, MA, USA), 10 ng/mL bFGF and 10 μM retinoic acid (Sigma-Aldrich)]. Upon eight days of differentiation, cells were removed by accutase (Corning) treatment and seeded as single cells on 24-well Transwells (polyester, 0.4 μm pore size; filter area 0.33 cm^2^, Corning) coated with a solution of collagen from human placenta (Sigma-Aldrich) and bovine plasma fibronectin (Sigma-Aldrich) (400 μg/mL collagen IV and 100 μg/mL fibronectin) at a density of 1,000,000 cells/cm^2^. Twenty-four h after seeding, EC–medium was added (EC medium supplemented with 1% platelet-poor derived serum). Purified endothelial monolayers were formed on day 10 of the experiment, and permeability barrier function tests were performed 48 h after seeding on the Transwell system.

### Measurement of barrier function

Barrier integrity of BMECs monolayer was obtained by measuring transendothelial electrical resistance (TEER) while using a Millicell ERS electrode (Millipore, Bedford, MA, USA). After conducting three measurements for each insert (n = 3), the average resistance was obtained. Paracellular permeability was assessed by adding 1 mg/mL of [^13^C_6_] mannitol and [^13^C_12_] sucrose to the donor site of the Transwell system. Then, 50 μL of aliquots were collected from the acceptor (basolateral chamber) at 10, 20, 30, 60, and 120 min. At the end of the experiment, the donor and acceptor samples were diluted in water to be in the range of standard curve (10–1000 ng/mL) and the aforementioned preparation steps were performed to measure the concentrations with UPLC–MS/MS system.

The clearance or permeability-surface area product (PS) for mannitol and sucrose were calculated using the following steps: First, the cleared volume up to each time point was calculated from the following equation. Then, linear regression applied to the plotted cleared volume versus time for samples and blank to obtain the PS of the Transwell system.

6 $$ {\text{Cleared Volume}} = \left( {C\left( {acceptor  } \right) *V\left( {acceptor} \right)} \right)/ C\left( {donor} \right) $$

Here, *C*_*acceptor*_ referred to measured concentration in acceptor compartment at a given sampling time point, and *V*_*acceptor*_ referred to the volume of acceptor compartment. Also, *C*_*donor*_ is the concentration in donor compartment. Afterwards, the permeability coefficient (P) was obtained by the following equations:

7 $$ {\text{P}} = {\text{PS}}/{\text{S}} $$

8 $$ \frac{1}{{  P_{Cells} }} = \frac{1}{{P_{total} }} - \frac{1}{{P_{blank} }} $$

The permeability coefficient (P) was obtained by dividing the PS to insert surface area (S) (cm^2^) Eq. (7), and then the permeability coefficient of the cell monolayer (P_*cells*_) was obtained by subtracting the permeability coefficient of Transwell (P_*total*_) from the permeability coefficient of the coated filter (P_*blank*_) Eq. (8).

### Measurement of permeability coefficient of Mannitol and Sucrose in presence of inflammatory cytokine

The effect of Interleukin 1 beta (IL-1β) on the permeability of mannitol and sucrose in in vitro model of BBB (iPSC-derived BMECs) was also measured. To mimic inflammatory conditions, the Transwell model was exposed to media supplemented with 10 and 100 ng/mL IL-1β (Peprotech, Cranbury, NJ) for 24 h (n = 3). Then, the medium was removed, and fresh medium containing 1 mg/mL of sucrose and mannitol was added to the apical side of the Transwell. The permeability coefficients of markers were measured as previously described. Also, the TEER values of iPSC-derived BMECs were measured before and after exposure to IL-1β.

### Measurement of partition coefficients of Mannitol and Sucrose

By using an established method, the partition coefficients of [^13^C_6_]mannitol and [^13^C_12_]sucrose between 1-octanol and water were determined [15]. For this purpose, an equal volume of 1-octanol and water were mixed together at room temperature overnight with continuous stirring. Then 100 μg/mL of [^13^C_6_]mannitol and [^13^C_12_]Sucrose were added to 5 mL saturated water, and then the mixture was added to 5 mL of saturated 1-octanol in a glass scintillation vial. Subsequently, the glass vial was placed in a rotary machine, and the content was mixed for 30 min. 500 μL samples were taken from both the water and 1-octanol phase for further analysis with LC-MS/MS. The water samples are diluted 100-fold, and the 1-octanol samples remain undiluted for this purpose.

### Statistical analysis

Statistical analysis of data was performed using Prism 8 (GraphPad Software, La Jolla, CA). All experimental metrics were collected across at least three biological replicates. The student’s paired t-test was used for comparison of uncorrected and corrected vascular space for the same mice. Unpaired two tailed t-test was used for comparison of two groups. Data with 3 groups were analyzed by 1-way ANOVA, followed by Tukey’s multiple comparison test. In all cases, a p value < 0.05 was considered significant. Data are presented as mean ± SD or individual values.

## Results

### Method development and validation

The mass spectra of [^13^C_12_], [^13^C_6_], and [^2^H_2_]sucrose have been reported in our previous study [13]. The best m/z transition of stable isotopes of mannitol was selected based on signal to noise ratio and higher sensitivity, see Fig. S1 in Additional file 1.

### Selectivity

**Fig. 1:**
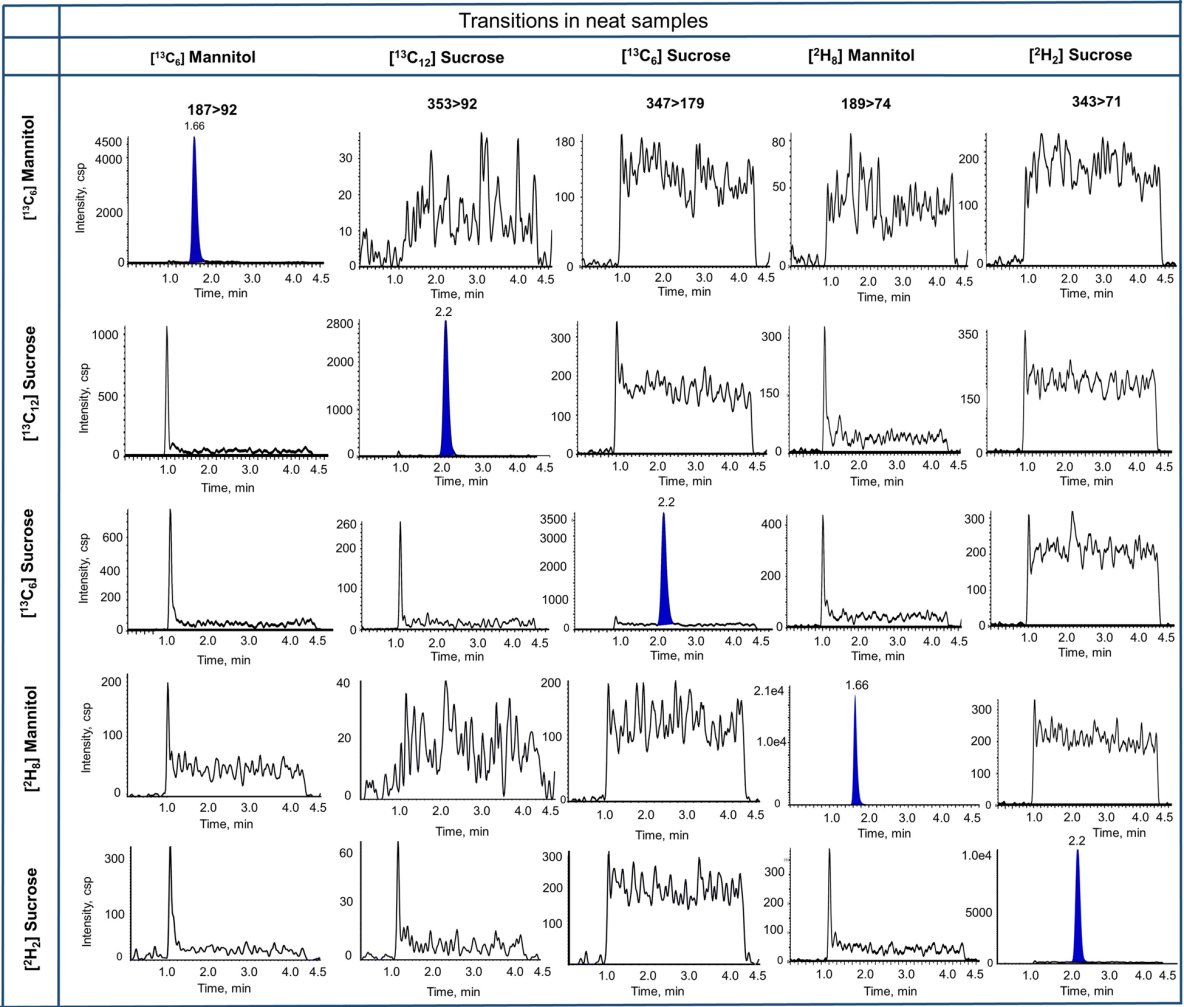
Chromatograms of single analyte neat samples of [^13^C_6_]mannitol, [^13^C_12_]sucrose, [^13^C_6_] sucrose, [^2^H_8_]mannitol, and [^2^H_2_]sucrose, prepared in LC–MS/MS grade water, with all considered transitions

**Fig. 2:**
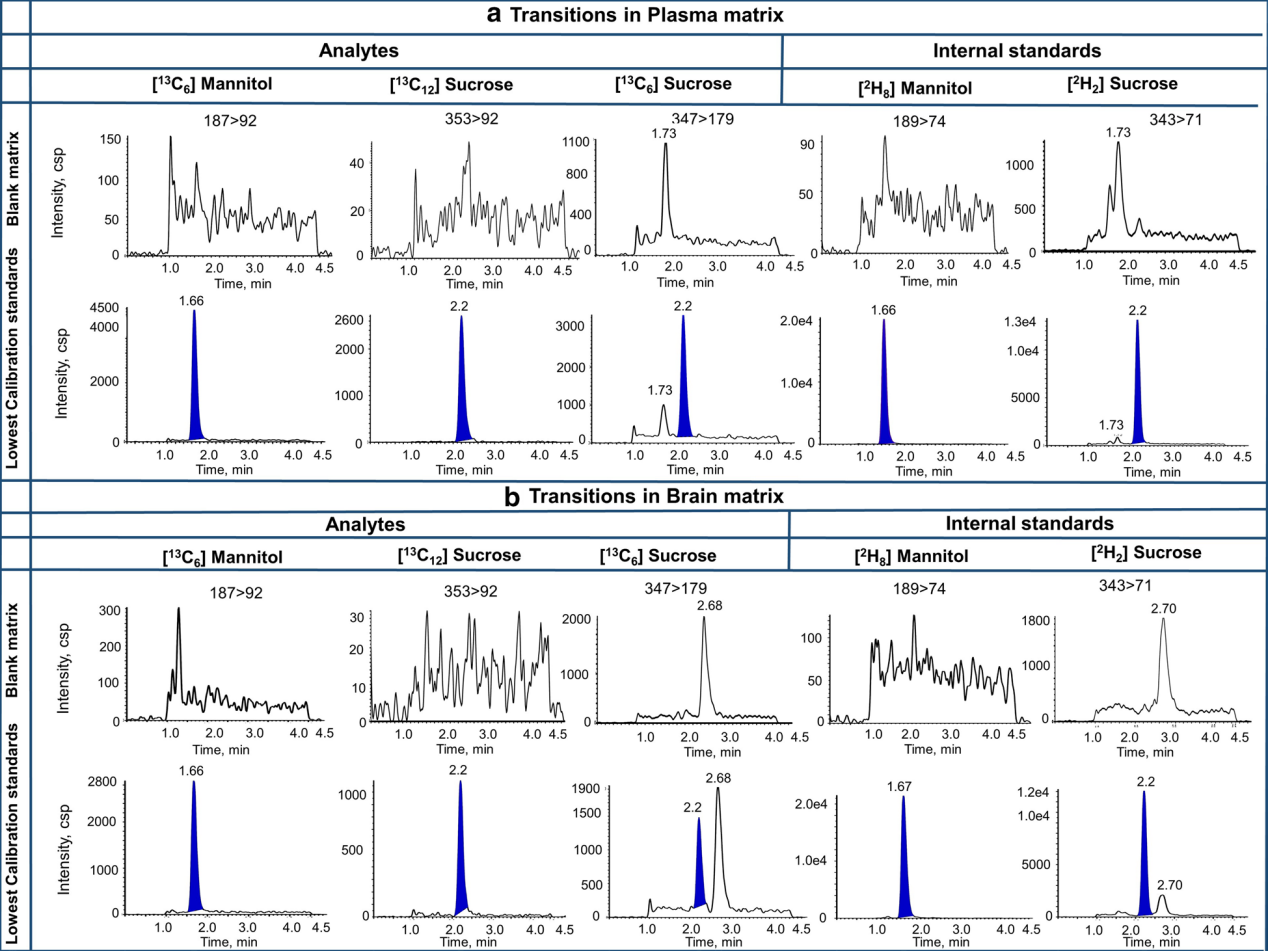
Chromatograms of blank matrices, Lowest calibration standard and internal standard in plasma (**a**) and brain matrix (**b**)

Figure 1 depicts the chromatograms of single analyte neat samples of mannitol and sucrose prepared in water with no cross channel interference between transitions observed. We also showed the lowest calibration standard, blank matrix and internal standard in plasma and brain matrix (Fig. 2). We found no interference in matrix samples. However, [^13^C_6_]sucrose (347 > 179) had a peak at retention time approx. 1.7 and 2.6 min in plasma and brain matrix, respectively. Also, [^2^H_2_]sucrose transition (343 > 71) displayed the same peaks at the retention time of 1.7 and 2.7 min for plasma and brain matrix, respectively. These peaks do not interfere with sucrose peak at the retention time of 2.2 min.

### Accuracy and precision

The data for Inter- and Intra-run accuracy and precision in plasma and brain samples are included in Additional file 1: Table S1 and S2. Both plasma and brain values were within the limits of Food and Drug Administration (FDA) guidelines for the method validation. Moreover, the calibration curves generated in the ranges of 10–1000 ng/mL and 5–400 ng/mL for plasma and brain, were found to be linear with r^2^ > 0.99 across all Intra and Inter-assay runs.

### Recovery and stability

Recoveries of [^13^C_6_]mannitol, [^13^C_12_]sucrose and [^13^C_6_]sucrose as the analytes of the method were performed for plasma and brain matrix at low, medium, and high concentrations. Based on Table S3, the recoveries of analytes were relatively high (≥ 95%) in all the tested matrices. Plus, the recovery of both sucrose analytes was similar to our previously developed method [13, 14]. In addition to high recovery, these data suggest minimal or no matrix effect on the analyte signal intensity.

The freeze–thaw stability of [^13^C_6_]mannitol was determined for 50 and 500 ng/ml in water (Additional file 1: Fig. S2). The results confirmed that mannitol stays stable at three cycles of freeze–thaw that was similar to sucrose analytes in our previous study [13]. Results from the long term storage stability also showed mannitol was stable in plasma and brain matrix over the long term. Regarding accuracy, the values of [^13^C_6_]mannitol in plasma at nominal concentrations of 10, 100, and 1000 ng/mL were 96.1%, 109%, and 97.6%, respectively. In case of brain matrix, the accuracy values for 5, 50 and 400 ng/mL nominal concentrations were 105%, 97.2%, and 97.5%, respectively.

### In vivo application of the method

**Fig. 3:**
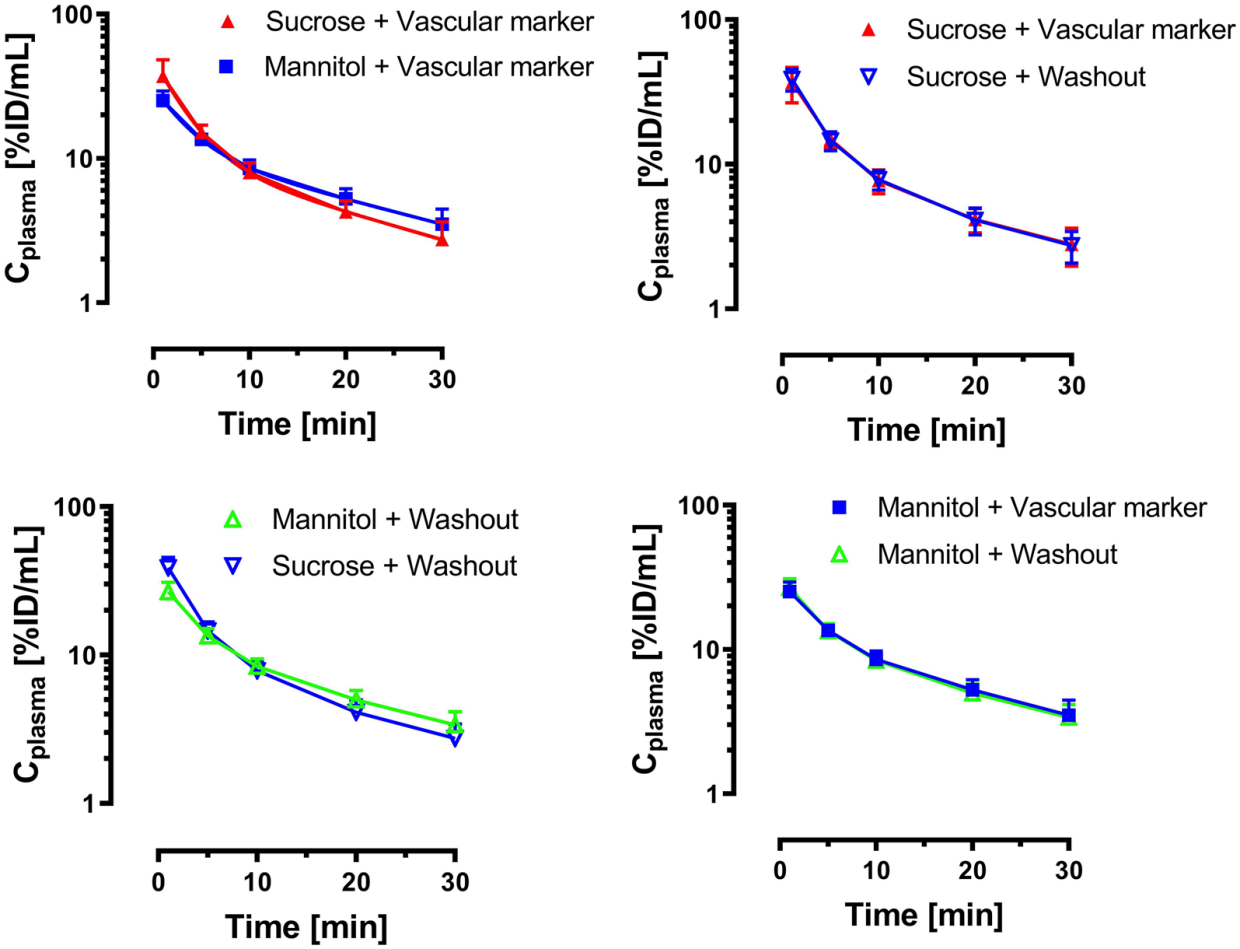
Pharmacokinetic profiles for [^13^C_6_]mannitol and [^13^C_12_]sucrose in mouse plasma up to 30 min after IV bolus (mean ± SD, n = 6)

**Fig. 4:**
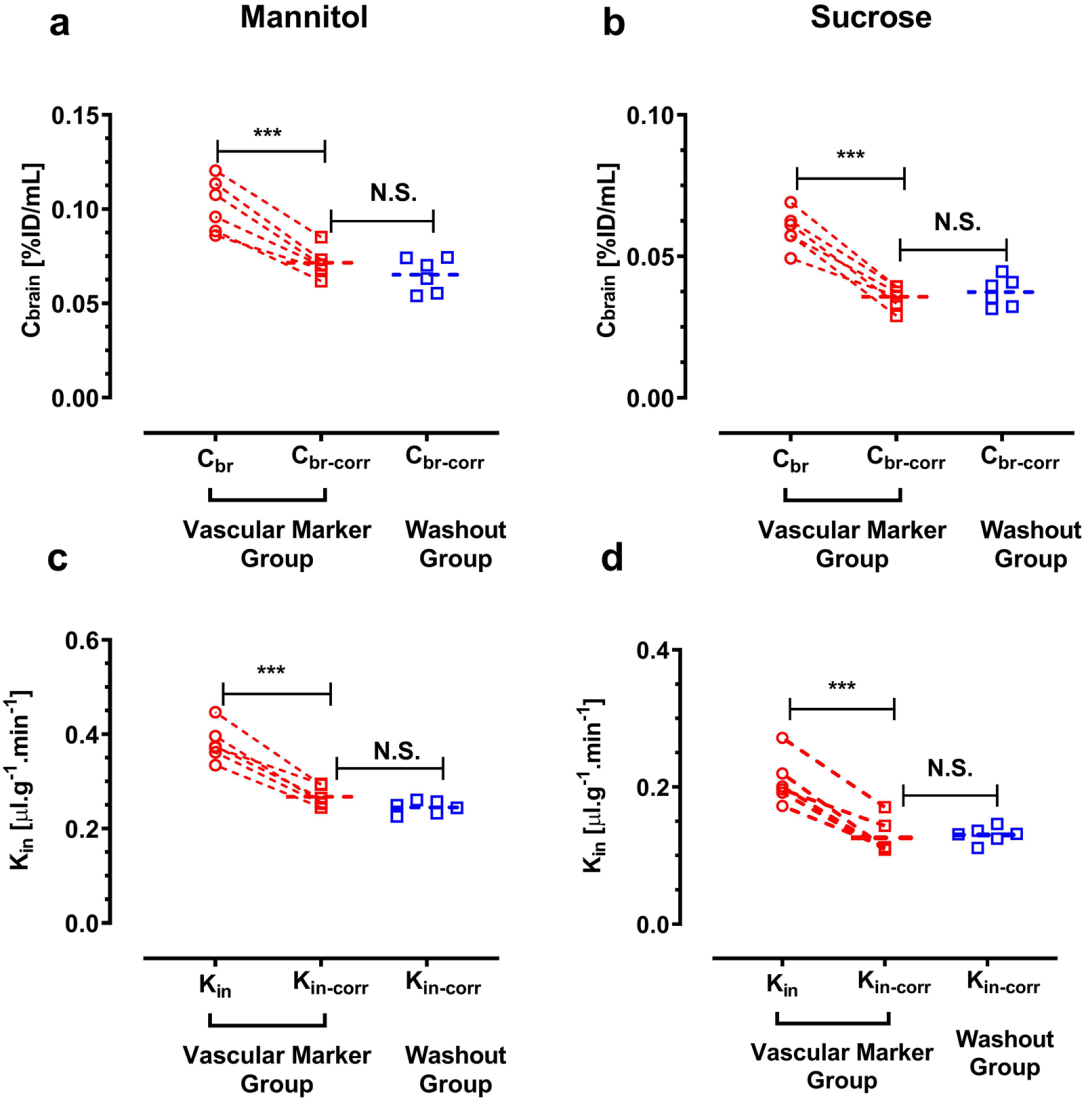
**a**, **c** Differences in brain concentration and brain uptake clearance (K_in_) of [^13^C_6_]mannitol with or without correction by vascular marker. **b**, **d** C_br_ and K_in_ of [^13^C_12_]sucrose with or without correction by vascular marker. ***p < 0.001 (n = 6), analyzed by Student’s paired t-test (two-tailed). N.S. Student’s unpaired t-test

A comparative pharmacokinetic study was done in two groups of anesthetized C57BL/6J mice to show the application of the method. The results of the pharmacokinetic study are shown in Figs. 3 and 4. The plasma profiles of both groups (vascular marker group and washout group) were similar for both mannitol and sucrose, and the areas under the curve from 0 to 30 min were not significantly different. Moreover, the plasma profile of mannitol was similar to sucrose, which showed a biexponential decline (Fig. 3).

Comparison of the corrected brain concentrations (washout vs. vascular marker correction) showed no significant difference for both mannitol and sucrose (unpaired, two-tailed t-test) (Fig. 4). C_br_ (%ID/mL) of mannitol was 0.071 ± 0.007 and 0.065 ± 0.009 for vascular marker and washout respectively, whereas the C_br_ of sucrose was almost half of mannitol C_br_ values (0.035 ± 0.003 and 0.037 ± 0.005 for vascular marker and washout respectively). Similarly, comparison of the brain uptake clearance (K_in_) between these two groups of each marker showed no significant difference (unpaired, two-tailed t-test) (Fig. 4). For example, The K_in_ value of mannitol was 0.267 ± 0.021 μL/min g^−1^ and 0.245 ± 0.013 μL/min g^−1^ for the vascular marker and washout groups, respectively. In terms of comparison of two markers, the K_in_ of mannitol (0.267 ± 0.021 µl g^−1^ min^−1^) was more than two times higher than that of sucrose (0.126 ± 0.025 µl g^−1^ min^−1^).

### In vitro application of the method (in vitro-in vivo correlation)

**Fig. 5:**
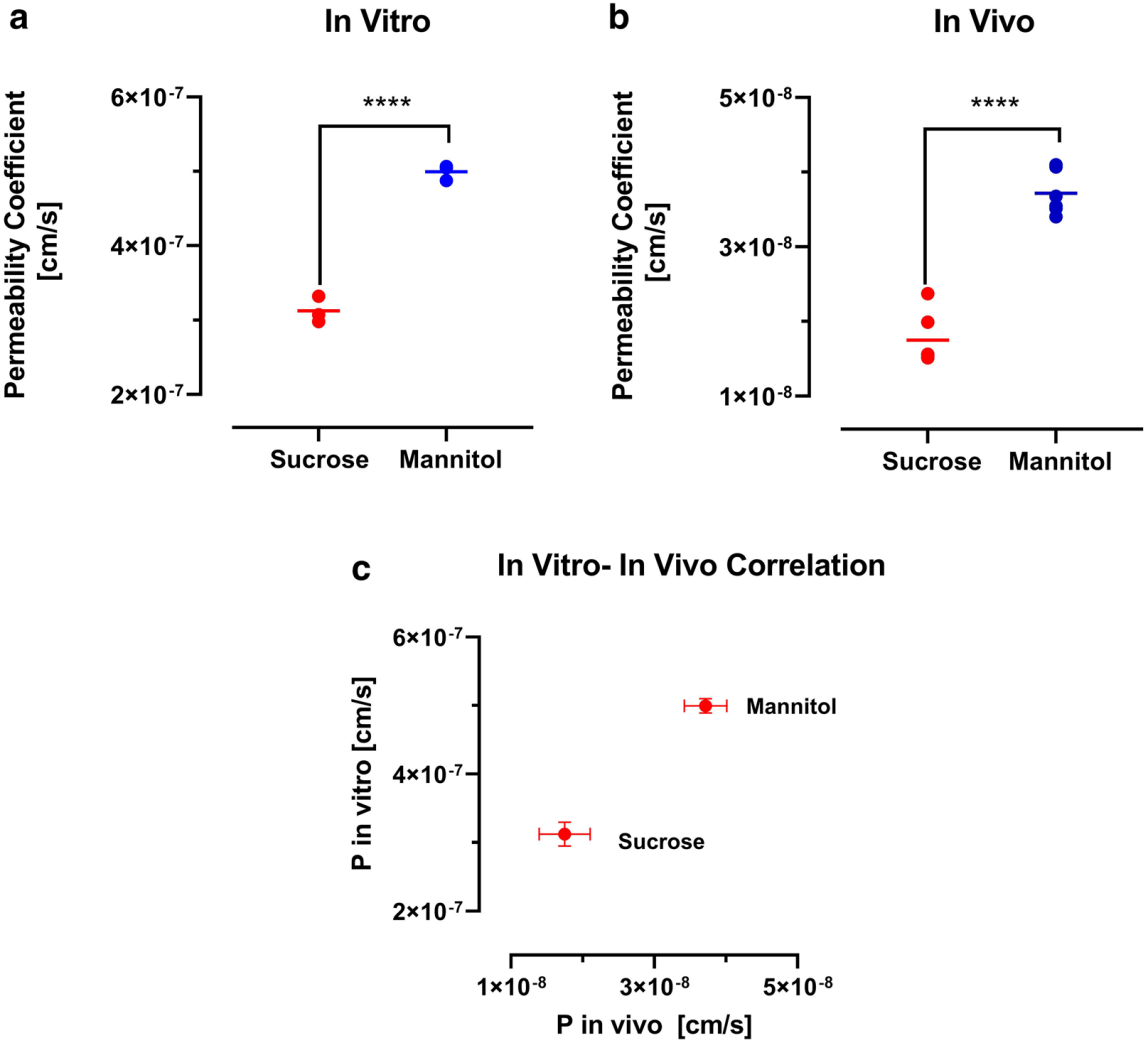
**a** Permeability coefficient (P) of mannitol and sucrose in the Transwell model with TEER value of 1812 ± 54 Ω cm^2^ (n = 3). **b** Permeability coefficient (P) of mannitol and sucrose in the vivo model (n = 6). ****p < 0.0001, analyzed by Student’s unpaired t-test (two-tailed). **c** In vitro and in vivo correlation of mannitol and sucrose based on the permeability coefficients

Transwell system is widely used in in vitro models of BBB for drug development and screening [26]. We evaluated the permeability of our novel markers in iPSC derived brain endothelial cells cultured on the Transwell membranes. The barrier function of the monolayer was confirmed by measuring the TEER. The average TEER value was 1812 ± 54 Ω cm^2^, which is similar to values reported in the literature [24, 27]. For a comparison between in vitro and in vivo models, the K_in_ values or permeability surface area products (PS) were converted to permeability coefficients, taking 0.33 cm^2^/well as the surface area of the Transwell membranes, and 120 cm^2^/g of brain as the surface area of the BBB in vivo [23]. The in vitro permeability coefficient of mannitol and sucrose was 4.99 ± 0.152 × 10^−7^ and 3.12 ± 0.176 × 10^−7^, respectively. Figure 5a depicts the permeability values of the two markers, with mannitol showing higher permeability compared to sucrose (p < 0.0001 unpaired, two-tailed t-test). The PS value of mannitol and sucrose in vivo was 0.267 ± 0.021 and 0.126 ± 0.025 µl g^−1^ min^−1^ respectively, which corresponds to a permeability coefficient value of 3.71 ± 0.296 × 10^−8^ and 1.75 ± 0.355 × 10^−8^ cm/s for mannitol and sucrose, respectively. Figure 5c showed the in vitro and in vivo correlation of the markers. Interestingly, the P values for mannitol and sucrose in vitro were only about 13-fold and 18-fold higher than the permeability coefficient in vivo.

**Fig. 6:**
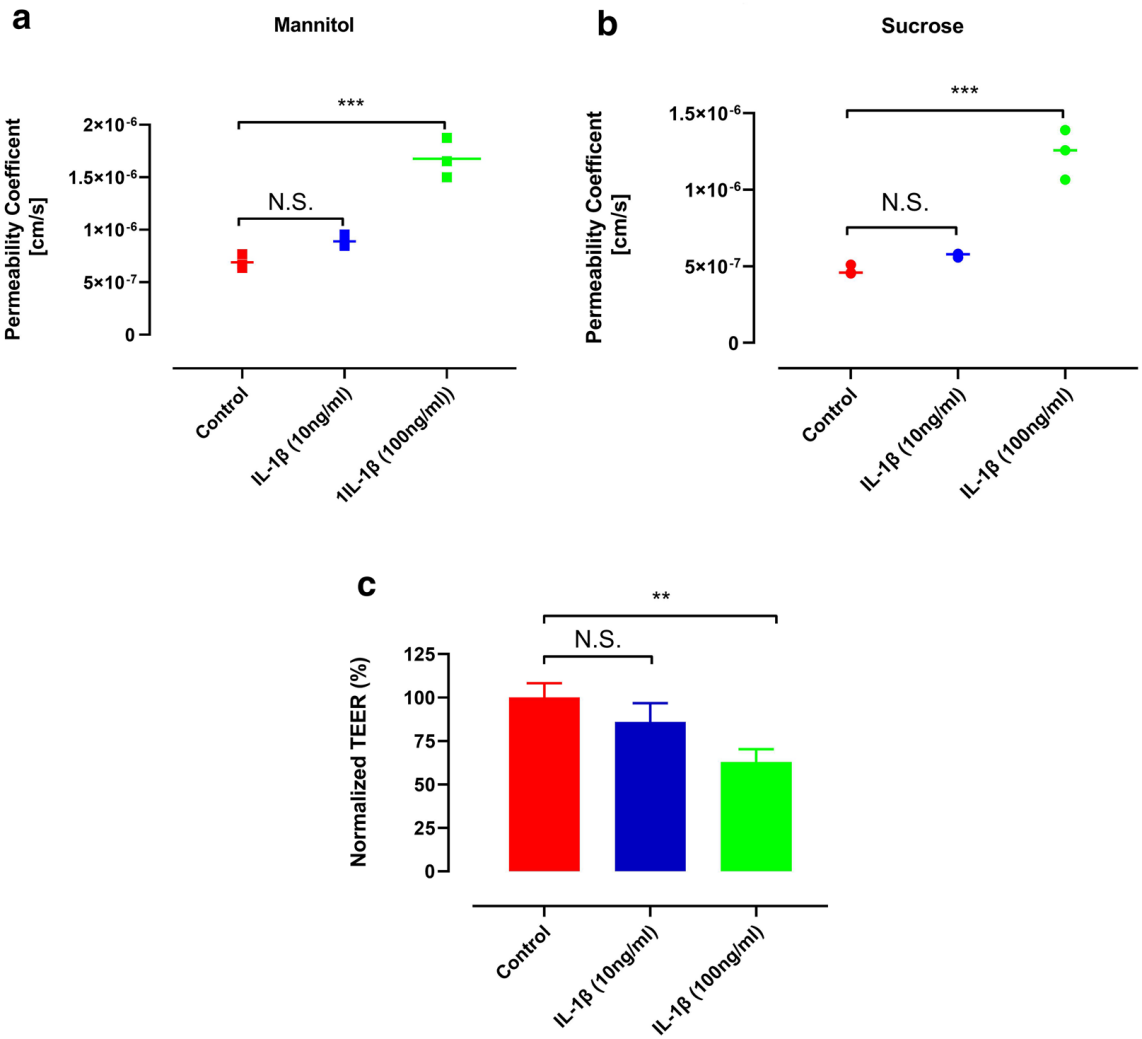
Permeability coefficient of **a** mannitol, **b** sucrose in iPSC-BMECs following treatment with different concentrations of IL-1β. **c** The effect of IL-1β cytokine on TEER of iPSC-BMECs. **p < 0.01 and ***p < 0.001, 1-way ANOVA, followed by Tukey’s multiple comparisons test (n = 3)

The effect of an inflammatory cytokine on the permeability of BBB in the vitro model was examined. As shown in Fig. 6, the permeability coefficient of mannitol and sucrose significantly increased from 6.90 ± 0.689 × 10^−7^ and 4.74 ± 0.314 × 1^0−7^ to 1.67 ± 0.188 × 10^−6^ and 1.23 ± 0.163 × 10^−6^, respectively, with 100 ng/mL IL-1β. Moreover, The TEER values of iPSC-derived BMECs decreased 38% after 1 day exposure to 100 ng/mL IL-1β. However, the decrease in the TEER values and increases in the permeability coefficients of sucrose and mannitol in the presence of 10 ng/mL IL-1β were not statistically significant (Fig. 6).

**Fig. 7:**
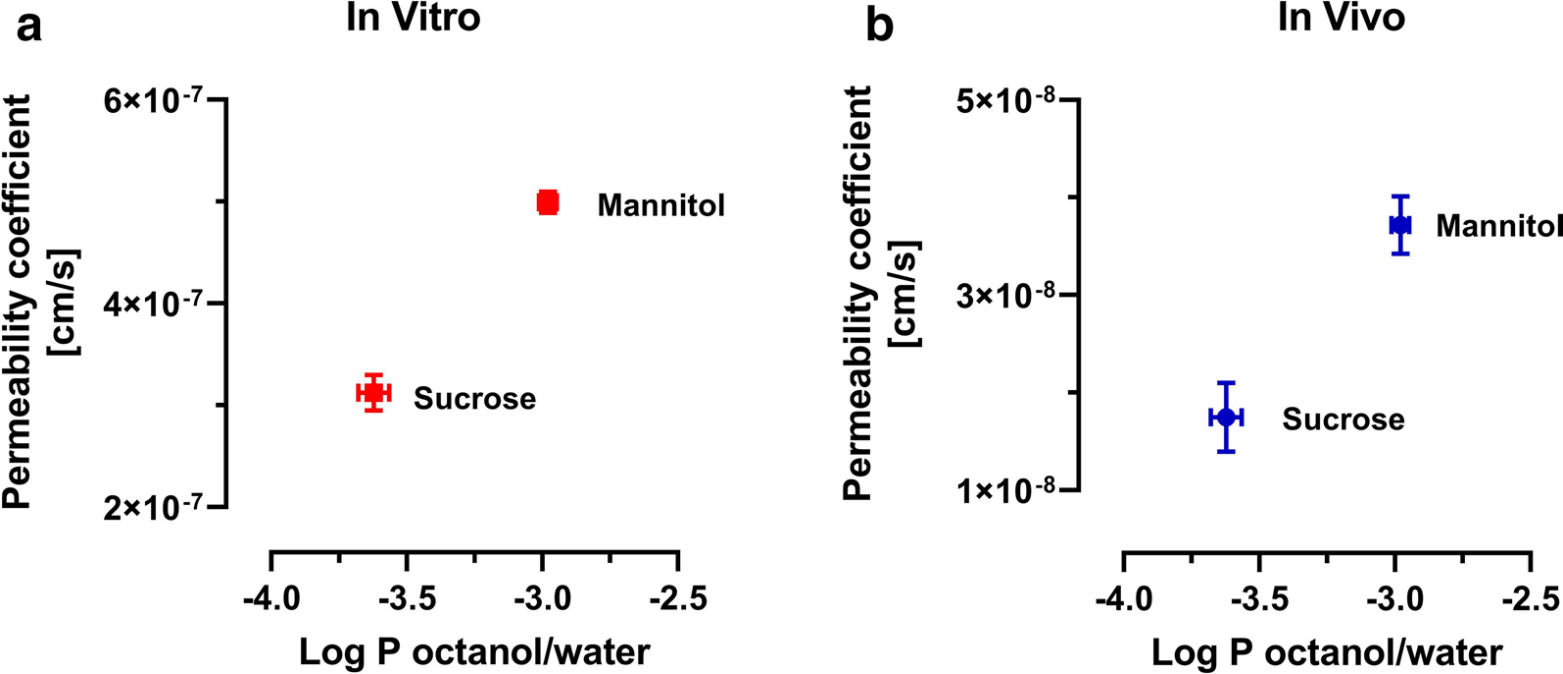
**a** Correlation of in vitro permeability coefficient (n = 3) and log P (n = 5). **b** Correlation of in vivo permeability coefficient (n = 6) and log P (n = 5)

The correlations between log P (partition coefficient) and in vitro and in vivo permeability coefficients are shown in Fig. 7. We found that mannitol and sucrose have a log P of − 2.98 ± 0.033 and − 3.62 ± 0.056. The permeability coefficient of sucrose is lower compared to mannitol, reflecting the lower log P value.

## Discussion

The results of our study showed we could accurately quantify the stable labeled isotopes of mannitol and sucrose simultaneously in brain and plasma by LCMS/MS. Our method not only could replace radioactive tracers of mannitol and sucrose for permeability studies, but also it could detect two different molecular weight markers by one single run. The radioactive version of mannitol has been used widely in the measurement of the BBB [17–20, 27]. Moreover, mannitol is also used as a marker in the lactulose/mannitol (L/M) ratio test as a widespread dual-sugar test to assess the intestinal barrier function [16]. [^13^C]mannitol has been recently presented as a novel biomarker for quantifying the intestinal permeability [28, 29] but, the validation of [^13^C]mannitol as a marker for the BBB has not been reported. Hence, the mass spectrometry condition of [^13^C_6_]mannitol was optimized by continuous injection of mannitol solution with injection pump to get the optimum M–H^−1^. According to our findings, the most robust m/z value for [^13^C_6_]mannitol was 187 → 92, based on signal to noise ratio.

The dual analytes with a vascular marker of sucrose were previously developed by our group. In our study, three analytes (one mannitol and two sucrose) plus two stable isotope internal standards were easily detected due to co-elution from a suitable stationary phase and variation of detector signal based on their molecular weight for each analyte. Figure 2 shows the simultaneous detectability of mannitol and sucrose in the various matrices. Co-administration of mannitol and sucrose could provide information on the uptake mechanism at the BBB of markers that have similar physicochemical properties over a range of molecular weights which covers the vast majority of marketed drugs [30]. The method has this feature to be run for single analyte by removing the transition of another anlayte from the method.

To indicate the application of our method in in vitro studies, we used the Transwell system as the in vitro platform most commonly used for BBB permeability studies. iPSC-derived BMECs were used, which provide high TEER values resulting in low paracellular permeability. The iPSC-derived BMECs is considered the ideal cell line for drug screening and permeability studies [31]. Various BBB permeability markers are currently being used for Transwell models and advanced microfluidic models, including sodium fluorescein, radiolabeled sucrose, and different molecular weight dextrans [32]. Our method can quantify with high sensitivity and accuracy the integrity of the BBB, when compared to radiolabeled versions of sucrose or mannitol, and the fluorescent dye sodium fluorescein, which all have drawbacks.

In terms of comparison between studies using radioactive versions of mannitol and sucrose measured by liquid scintillation counting with their stable isotopes analyzed by LC–MS/MS, we have previously shown that [^14^C]sucrose had a 6 to sevenfold higher K_in_ value in vivo than [^13^C_12_]sucrose [15]. We also found by chromatographic fractionation of [^14^C]sucrose after in vivo administration that the majority of the brain content of measured ^14^C radioactivity belonged to compounds other than the intact [^14^C]sucrose [15]. Here, we found the K_in_ of mannitol (0.267 ± 0.021) 3–7 fold lower than published values obtained with radioactive versions of mannitol ([^14^C] and [^3^H] mannitol) [18, 19, 33, 34]. Moreover, Preston and Haas reported 30–40% lower permeability area products of chromatographically purified [^3^H]mannitol compared to stock solution of the same tracer lot [33, 35]. Comparing the permeability values in different in vitro studies is challenging due to major differences in experimental design in the published studies, including different sources of the endothelial cells and different culture conditions, which also results in a range of different TEER values. Recent iPSC-derived BMECs in vitro models reported similar permeability values (in the range of 10^−6^ to 10^−7^ cm/s) for mannitol and sucrose as our values [24, 36].

With respect to the small molecule fluorescent dye marker, sodium fluorescein, we have shown in previous work that, in order to avoid erroneous interpretation of brain uptake data, it is mandatory to perform a chromatographic analysis of the unmetabolized (non-glucuronidated) substance, and to measure the free fraction in plasma [37, 38]. Both is often neglected in publications using fluorescein in studies on BBB permeability. In addition, the potential role of efflux transporters for fluorescein at the BBB has not been conclusively ruled out [39–41].

Recent advanced microfluidic BBB models (BBB-on-a-chip) have reported general barrier restrictiveness by measuring paracellular flux with different molecular weights of dextran (ranging from 3 to 70 kDa). Reporting barrier function for large molecular weight markers may not accurately predict the integrity of BBB models for small, drug like molecules [42, 43]. Furthermore, permeability quantifications with such markers are not reliable in in vivo experiments and result in inaccurate comparison between these advanced in vitro models and in vivo models. We obtained a permeability coefficient of 4.99 ± 0.152 × 10^−7^ and 3.12 ± 0.176 × 10^−7^ for mannitol and sucrose, respectively. The permeability was found to be very low and showed the human in vitro model has very tight barrier properties. Moreover, the precision and accuracy of the method supports its use for in vitro-in vivo correlation studies with respect to permeability properties under healthy and disease conditions.

We also found that 100 ng/mL IL-1β resulted in a change of barrier function in the in vitro model. This observation is similar to the previous in vitro reports [44–46]. Additionally, there was a trend towards a decrease in the TEER value accompanied by an increase in the permeability coefficients for both markers at the 10 ng/mL concentration of IL-1β. However, these changes did not reach statistical significance (Fig. 6), most likely due to the small sample size used in our study (n = 3). The developed LC–MS/MS method was successfully applied to measurements of plasma, and brain concentrations of mannitol and sucrose after injection of the markers to mice at a dose of 10 mg/kg. We previously showed that correcting vascular space using [^13^C_6_]sucrose was equally effective as buffer perfusion for determination of the BBB permeability of [^13^C_12_]sucrose [13]. Interestingly, similar results were obtained when we used [^13^C_6_]sucrose for correcting the vascular space of mannitol analyte in the brain (Fig. 4). The corrected K_in_ and C_br_ of analytes showed no significant difference between the vascular marker and washout groups for both mannitol and sucrose. However, the uncorrected K_in_ and concentration of brain indicated an overestimation, almost two times higher than the correct values, which demonstrates the impact of the intravascular content. In this context it is also apparent that correction of intravascular volume needs to be performed in each individual animal, rather than by a value determined in a separate experimental series.

The correction method by vascular marker administration could be practically more advantageous compared to the washout method in several aspects: Technically, it is easier to perform, and brain tissue collection is attainable within seconds after the terminal blood sampling, as opposed to delays for several minutes by performing thoracotomy and perfusion (e.g., over 10 min in the present study). Furthermore, rapid sampling gains importance when, apart from measuring the BBB permeability, parts of the brain samples were needed for measurement of other analytes such as neurotransmitters or metabolites, that may undergo rapid degradation.

By comparing the PK profile of the two markers, we found that the plasma profiles of mannitol and sucrose were similar. However, the brain concentrations and K_in_ of mannitol were almost two-fold higher than those for sucrose, which could be related to its lower molecular weight and higher paracellular diffusibility. An alternative explanation is the slightly higher lipid solubility of mannitol, with a log P of − 2.98 ± 0.033, which is half a log order higher than that of sucrose − 3.62 ± 0.055.

## Conclusion

In conclusion, the newly developed method allows the measurement of triple analytes of mannitol and sucrose in the same sample in a single run. This technique simplifies correction for intravascular plasma space in brain uptake experiments with sucrose or mannitol and makes a vascular washout step dispensable. In addition, non-radiolabeled [^13^C_6_]mannitol was introduced as BBB marker for the first time in this study. Last but not least, this method can now be considered as a very useful tool in quantifying BBB permeability in different in vitro and in vivo disease models as well as for monitoring treatment outcomes.

## Supplementary information

**Additional file 1. Figure S1.** Mass spectra of [^13^C_6_]mannitol and [^2^H_8_]mannitol. **Figure S2.** Freeze thaw stability of [^13^C_6_] mannitol. **Table S1.** Inter-run and Intra-run accuracy and precision values of analytes for plasma. **Table S2.** Inter-run and Intra-run accuracy and precision values of analytes for brain. **Table S3.** Recoveries of analytes in plasma and brain matrix.

## Data Availability

All data generated or analyzed during this study are included in this published article.

## Abbreviations

BBB: Blood brain barrier
UPLC: Ultra performance liquid chromatography
LC-MS: Liquid chromatography and mass spectrometry
iPSC: Induced pluripotent stem cells
BMECs: Brian microvascular endothelial cells
CNS: Central nervous system
