# Supplementary material for: LC–MS/MS-based in vitro and in vivo investigation of blood–brain barrier integrity by simultaneous quantitation of mannitol and sucrose
Source: Fluids Barriers CNS. 2020 Oct 14;17:61. doi: 10.1186/s12987-020-00224-1 (PMC7556948; doi:10.1186/s12987-020-00224-1)
Supplement: Supplementary file 1 — Additional file 1. Figure S1. Mass spectra of [13C6]mannitol and [2H8]mannitol. Figure S2. Freeze thaw stability of [13C6] mannitol. Table S1. Inter-run and Intra-run accuracy and precision values of analytes for plasma. Table S2. Inter-run and Intra-run accuracy and precision values of analytes for brain. Table S3. Recoveries of analytes in plasma and brain matrix. [file 12987_2020_224_MOESM1_ESM.docx]

**LC-MS/MS-based in vitro and in vivo investigation of blood-brain barrier integrity by simultaneous quantitation of mannitol and sucrose**

Behnam Noorani^1,4^, Ekram Ahmed Chowdhury^1,4^, Faleh Alqahtani ^2^, Yeseul Ahn^1,4^, Dhavalkumar Patel^1^, Abraham Al-Ahmad^1,4^, Reza Mehvar^3^, Ulrich Bickel^1,4^

**Additional information**

**Figure S1:** Mass spectra of [^13^C_6_]mannitol and [^2^H_8_]mannitol

**Figure S2:** Freeze thaw stability of [^13^C_6_] mannitol

**Table S1:** Inter-run and Intra-run accuracy and precision values of analytes for plasma

**Table S2:** Inter-run and Intra-run accuracy and precision values of analytes for brain

**Table S3:** Recoveries of analytes in plasma and brain matrix.


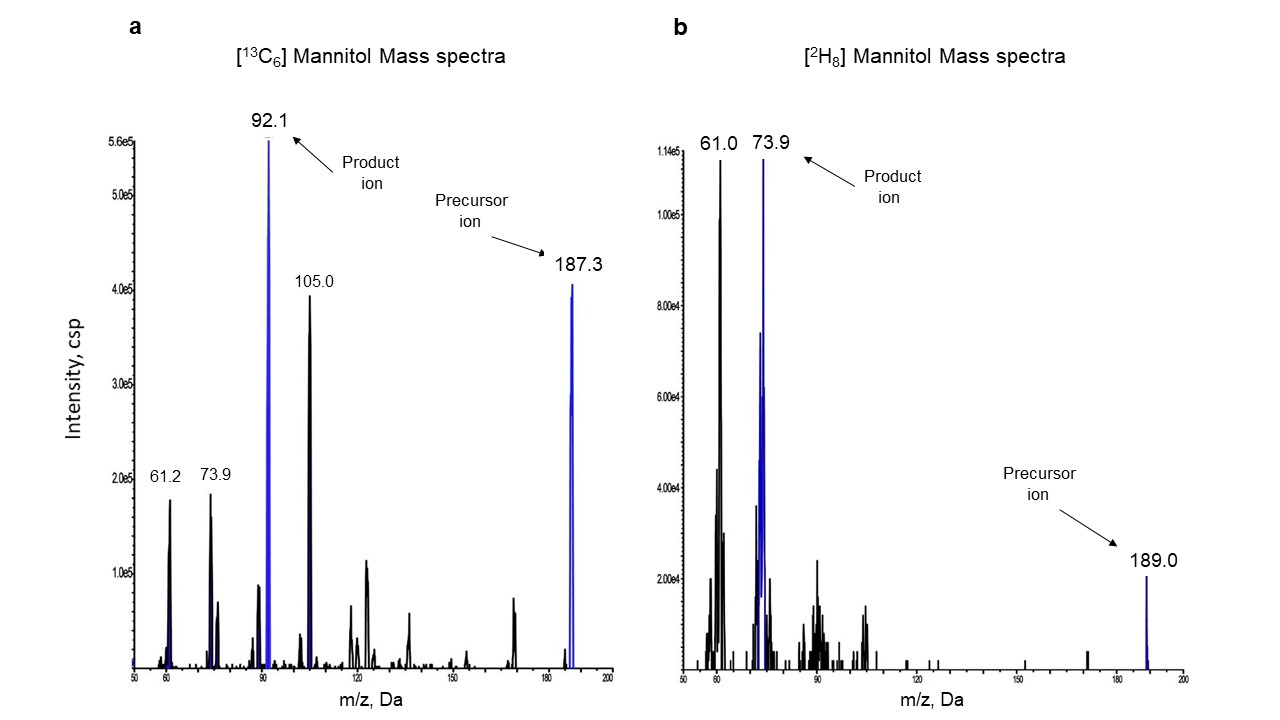


**Figure S1:** Mass spectra of a) [^13^C_6_]mannitol and b) [^2^H_8_]mannitol, demonstrating precursor and product ions considered in the validation. The best m/z transition of stable isotopes of mannitol was selected based on signal to noise ratio and higher sensitivity. The best m/z transition of stable isotopes of mannitol was selected based on signal to noise ratio and higher sensitivity. The transition 187 > 92 of [^13^C_6_]mannitol was found to be the best in terms of signal to noise ratio and sensitivity compared to other transitions ( 187>61 and 187> 73). In case of [^2^H_8_]mannitol, the m/z transition of 189> 73 was selected for our study, but the m/z transition of 189 > 61 is also a viable alternative due to the same signal to noise ratio and sensitivity.


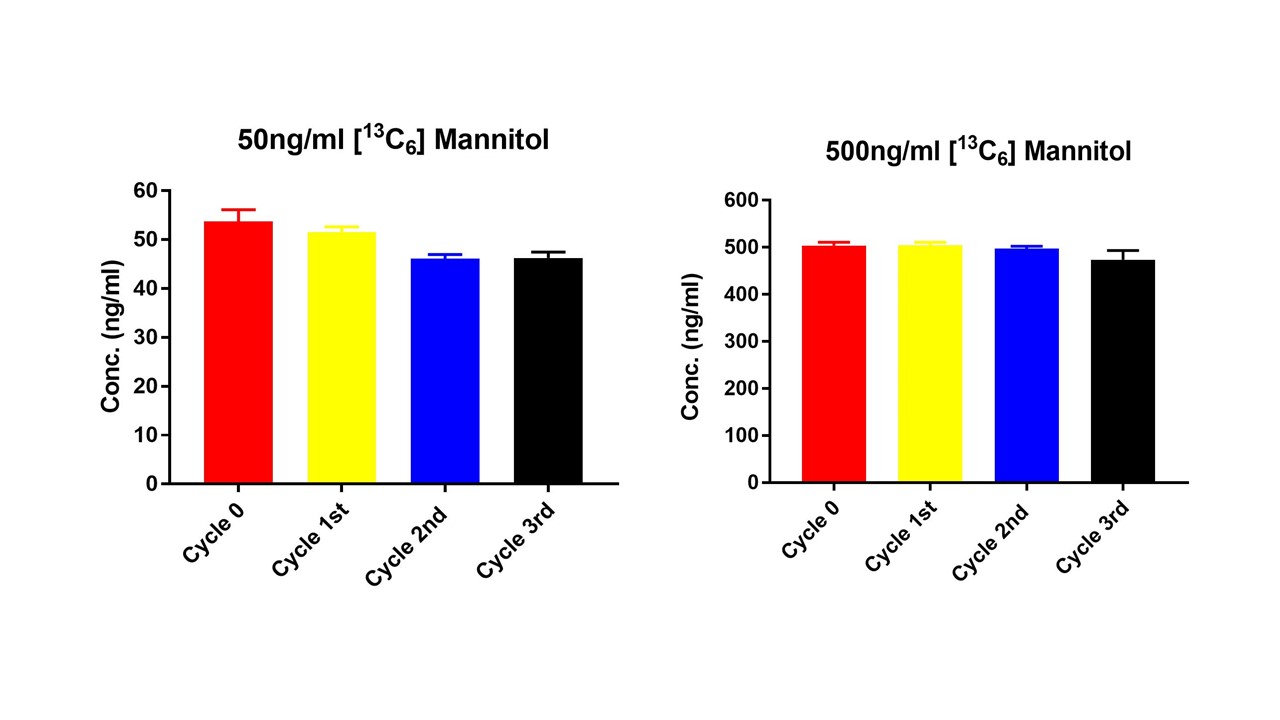


**Figure S2:** Freeze thaw stability of [^13^C_6_] mannitol (n =3). The freeze-thaw stability was performed by subjecting two neat concentrations of analytes (50 and 500 ng/mL) to three freeze/thaw cycles (n=3). Prepared samples were stored at −80 °C and thawed at room temperature for one hour, in order to replicate the experimental conditions. The concentration of the analytes in the neat samples was compared to the standard curve.

**Table S1:** Inter-run and Intra-run accuracy and precision values of the lowest, middle, and highest concentrations of triple analytes in standard curves for plasma (n=5). The accuracy was calculated as a percentage of measured concentration over nominal concentration. Precision was calculated as a percentage of relative standard deviations (RSD). The acceptable inter and intra-run limits for the accuracy were set at 85–115% for the middle and high concentrations and 80–120% for the low concentration. The standard precision values were 15% (medium and high concentrations) or 20% (low concentration).

| **Analyte** | **Added Conc. (ng/ml)** | **Interday run for plasma** | | |  | **Intra day run of plasma** | | |
| --- | --- | --- | --- | --- | --- | --- | --- | --- |
|  |  | **Measured Conc.**  **(ng/ml)** | **Accuracy (%)** | **Precision**  **(RSD %)** |  | **Measured Conc. (ng/ml)** | **Accuracy (%)** | **Precision**  **(RSD %)** |
| **[^13^C_6_] Mannitol** | 10 | 9.66 | 96.6 | 2.92 |  | 9.67 | 96.7 | 3.23 |
|  | 100 | 101 | 101 | 1.46 |  | 107 | 107 | 1.86 |
|  | 1000 | 938 | 93.8 | 3.34 |  | 982 | 98.2 | 2.83 |
| **[^13^C_12_]sucrose** | 10 | 10.1 | 101 | 7.58 |  | 10.7 | 107 | 2.12 |
|  | 100 | 101 | 101 | 3.34 |  | 99.6 | 99.6 | 2.17 |
|  | 1000 | 992 | 99.2 | 2.94 |  | 1000 | 100 | 1.99 |
| **[13C6]sucrose** | 10 | 10.3 | 100 | 5.52 |  | 10.3 | 103 | 2.97 |
|  | 100 | 100 | 100 | 2.86 |  | 102 | 102 | 1.73 |
|  | 1000 | 987 | 98.7 | 3.32 |  | 1010 | 101 | 1.56 |

**Table S2:** Inter-run and Intra-run accuracy and precision values of the lowest, middle, and highest concentrations of triple analytes in standard curves for brain homogenate (n=5)

| **Analyte** | **Added Conc. (ng/ml)** | **Interday run for Brain** | | |  | **Intra day run for Brain** | | |
| --- | --- | --- | --- | --- | --- | --- | --- | --- |
|  |  | **Measured Conc.**  **(ng/ml)** | **Accuracy (%)** | **Precision**  **(RSD %)** |  | **Measured Conc. (ng/ml)** | **Accuracy (%)** | **Precision**  **(RSD %)** |
| **[^13^C_6_] Mannitol** | 5 | 5.00 | 100 | 3.41 |  | 5.20 | 104 | 4.75 |
|  | 50 | 50.5 | 101 | 1.84 |  | 51.1 | 102 | 4.42 |
|  | 400 | 389 | 97.3 | 2.26 |  | 407 | 102 | 1.58 |
| **[^13^C_12_]sucrose** | 5 | 4.85 | 97.0 | 2.75 |  | 5.07 | 101 | 3.92 |
|  | 50 | 51.2 | 102 | 5.08 |  | 52.0 | 104 | 5.33 |
|  | 400 | 425 | 106 | 4.42 |  | 423 | 106 | 4.24 |
| **[13C6]sucrose** | 5 | 5.08 | 102 | 8.71 |  | 4.15 | 83.0 | 10.5 |
|  | 50 | 49.8 | 99.6 | 4.16 |  | 51.7 | 103 | 5.39 |
|  | 400 | 399 | 99.8 | 4.49 |  | 434 | 108 | 4.10 |

**Table S3:** Recoveries (mean ± SD) of analytes in a) plasma and b) brain matrix at low, medium and high concentrations(n = 5). Recovery was calculated as the percent of the ratio of peak areas Sample/Reference, where sample refers to the matrix and reference to water (neat sample), respectively.

| **Added Conc. (ng/ml)** | 1. **Analyte (Plasma recovery (%)± SD)** | | |
| --- | --- | --- | --- |
|  | **[^13^C_6_] Mannitol** | **[^13^C_12_] sucrose** | **[^13^C_6_] sucrose** |
| 10 | 102 ± 3.08 | 96.7 ± 2.25 | 100 ± 3.41 |
| 100 | 102 ± 3.72 | 97.3 ± 1.31 | 99.8 ± 1.76 |
| 100 | 96.4 ± 2.96 | 99.2 ± 2.57 | 98.8 ± 3.10 |
| **Added Conc. (ng/ml)** | 1. **Analyte (Brain recovery(%)± SD)** | | |
|  | **[^13^C_6_] Mannitol** | **[^13^C_12_] sucrose** | **[^13^C_6_] sucrose** |
| 5 | 107 ± 5.98 | 101 ± 4.57 | 94.7 ± 1.53 |
| 50 | 98.2 ± 4.00 | 102 ± 2.00 | 102 ± 2.66 |
| 400 | 94.8 ± 4.12 | 104 ± 4.58 | 103 ± 2.17 |
